# *Schistosoma japonicum* Soluble Egg Antigens Attenuate Invasion in a First Trimester Human Placental Trophoblast Model

**DOI:** 10.1371/journal.pntd.0002253

**Published:** 2013-06-06

**Authors:** Emily A. McDonald, Jennifer F. Friedman, Surendra Sharma, Luz Acosta, Sunthorn Pond-Tor, Ling Cheng, Eric S. White, Jonathan D. Kurtis

**Affiliations:** 1 Center for International Health Research, Rhode Island Hospital, Brown University Medical School, Providence, Rhode Island, United States of America; 2 Department of Pathology and Laboratory Medicine, Rhode Island Hospital, Brown University Medical School, Providence, Rhode Island, United States of America; 3 Department of Pediatrics, Rhode Island Hospital, Brown University Medical School, Providence, Rhode Island, United States of America; 4 Department of Pediatrics, Women and Infants Hospital, Brown University Medical School, Providence, Rhode Island, United States of America; 5 Department of Immunology, Research Institute of Tropical Medicine, Manila, The Philippines; 6 Department of Medicine, University of Michigan Medical School, Ann Arbor, Michigan, United States of America; Centers for Disease Control and Prevention, United States of America

## Abstract

**Background:**

Schistosomiasis affects nearly 40 million women of reproductive age, and is known to elicit a pro-inflammatory signature in the placenta. We have previously shown that antigens from schistosome eggs can elicit pro-inflammatory cytokine production from trophoblast cells specifically; however, the influence of these antigens on other characteristics of trophoblast function, particularly as it pertains to placentation in early gestation, is unknown. We therefore sought to determine the impact of schistosome antigens on key characteristics of first trimester trophoblast cells, including migration and invasion.

**Methods:**

First trimester HTR8/SVneo trophoblast cells were co-cultured with plasma from pregnant women with and without schistosomiasis or schistosome soluble egg antigens (SEA) and measured cytokine, cellular migration, and invasion responses.

**Results:**

Exposure of HTR8 cells to SEA resulted in a pro-inflammatory, anti-invasive signature, characterized by increased pro-inflammatory cytokines (IL-6, IL-8, MCP-1) and TIMP-1. Additionally, these cells displayed 62% decreased migration and 2.7-fold decreased invasion *in vitro* after treatment with SEA. These results are supported by increased IL-6 and IL-8 in the culture media of HTR8 cells exposed to plasma from *Schistosoma japonica* infected pregnant women.

**Conclusions:**

Soluble egg antigens found in circulation during schistosome infection increase pro-inflammatory cytokine production and inhibit the mobility and invasive characteristics of the first trimester HTR8/SVneo trophoblast cell line. This is the first study to assess the impact of schistosome soluble egg antigens on the behavior of an extravillous trophoblast model and suggests that schistosomiasis in the pre-pregnancy period may adversely impact placentation and the subsequent health of the mother and newborn.

## Author Summary

Approximately 40 million women of childbearing age suffer from schistosome infection globally at any given time. Multiple studies in rodent models, as well as a few reports in humans, suggest that schistosome infection results in poor pregnancy outcomes. We have previously shown that antigens released from schistosome eggs result in a pronounced pro-inflammatory response in syncytialized third trimester trophoblasts. Herein, we examine the effect of schistosome egg antigens on a first trimester trophoblast cell line, an accepted model for early placental development. Not only is the pro-inflammatory response recapitulated in this model system, but we also observed a decrease in migration and invasion of trophoblast cells after exposure to these antigens. Both migration and invasion are key aspects in early placental development, and inadequate invasion has been implicated in pregnancy-related diseases such as growth restriction and preeclampsia. This study is the first to examine the impact of schistosome antigens on early placental development, and may have implications for the subsequent health of both the pregnancy and the child.

## Introduction

Schistosomes are parasitic worms endemic to many parts of Africa, South America and Southeast Asia. They represent a significant disease burden in endemic regions, and have been estimated to be responsible for as many as 13–15 million disability-adjusted life years (DALYs) lost per year, with the true number potentially much higher [1]. Of the estimated 200 million people worldwide infected at any one time, approximately 40 million are women of reproductive age [2], [3]. A 2002 World Health Organization policy statement recommended the use of praziquantel in pregnant and lactating women [4], however many women still experience multiple cycles of pregnancy and lactation with schistosomiasis. This is due to the fact that in many regions of the world, pregnant and lactating women are still routinely excluded from treatment initiatives due to the Federal Drug Administration Class B designation that praziquantel still carries, as well as barriers to praziquantel acquisition and distribution.

Data from our laboratory and others have demonstrated poor reproductive outcomes in rodents and humans in the context of schistosomiasis. In rodent models, schistosome infection has profound impacts on birthweight and litter size [5]–[7]. We have previously shown that schistosome infection in a population of pregnant women residing in The Philippines is positively associated with increased risk for chorioamnionitis and increased pro-inflammatory cytokines in both maternal and cord blood [8]. Although very few adult worms and/or eggs are thought to directly traffic to the tissues of the maternal-fetal interface [9], [10], the residency of adult worms in the mesenteric vasculature and lodging of eggs in the liver allow for continuous secretion of antigens directly into the blood stream of the host. These antigens are known to traffic to, and cross, the human placenta, and have been found in fetal circulation [11]–[17]. We hypothesized that these antigens may have a direct effect on the cells at the maternal-fetal interface, and in the studies described herein, have chosen to focus specifically on processes specific to extravillous trophoblast cells.

Many events occur early in gestation that can have profound effects on the subsequent health of the fetus from gestation into adulthood. A lack of data pertaining to schistosomiasis during this critical window of development prompted us to utilize the first trimester cell line, HTR8/SVneo, to investigate the influence of schistosome infection on early events of pregnancy. Initial investigation was performed with co-culture of HTR8 cells and plasma collected from pregnant women infected with schistosomiasis or matched controls. To isolate the schistosome soluble egg antigen (SEA) specific effect on trophoblasts from any contribution of the host response, we next evaluated the direct impact of SEA on HTR8 cells. Events critical to placentation, including cytokine production, cellular migration and invasion were all assessed in an *in vitro* setting.

## Materials and Methods

### Ethics statement

For the HTR8 human plasma co-culture experiment, written informed consent was obtained from each participant, and the study was approved by the institutional review boards at Rhode Island Hospital and the Philippines Research Institute of Tropical Medicine.

### Cell culture

We used the immortalized first trimester cell line HTR8/SVneo, originally obtained from a human pregnancy terminated in the first trimester, and displaying properties of invasive extravillous cytotrophoblast cells [18]. Cells were maintained at 37°C with 5% CO_2_ in 1∶1 DMEM/F-12 media (Invitrogen, Grand Island, NY) supplemented with 1% L-glutamine (Invitrogen), 1% penicillin/streptomycin (Invitrogen) and 5% fetal bovine serum (Atlanta Biologicals, Lawrenceville, GA). All experiments were performed with the addition of SEA (25 µg/ml) in complete media for 24 h or media only control, unless otherwise noted. This dose was chosen based on dose response curves performed previously in our laboratory using purified primary trophoblast cells [19].

### Antigen preparation

Schistosoma *japonicum* SEA was generously donated by Dr. Chuan-Xin Yu (Jiangsu Provincial Institute of Schistosomiasis Control, Wuxi, Jiangsu, China) after having been prepared according to standard procedure [20]. The SEA was prepared under endotoxin free conditions, with all reagents and equipment used to isolate the SEA from collected livers being LPS free prior to use. Preparations were evaluated for contaminating endotoxin using an FDA-cleared LAL-assay (Lonza Group, Basel, Switzerland). Endotoxin levels for all SEA preparations used were <6 EU/mg protein, which, in our culture conditions, is at least 1000-fold lower than levels that have been previously shown to influence human trophoblast cells [21].

### Cytokine assays

Following the treatment period, media from HTR8/SVneo cells was collected and levels of multiple cytokines, chemokines, and fibrotic markers were assessed on a bead-based platform (BioPlex, Bio-Rad, Hercules, CA) using a sandwich antibody-based assay as previously described [22]. Cytokines evaluated included interleukin (IL)-1β, IL-6, interferon (IFN)-γ, tumor necrosis factor (TNF)-α, IL-4, IL-5, IL-10, IL-13, IL-12, IL-8 and IL-2. These specific cytokines were measured, as they were all components of a multiplex analysis developed and validated in our laboratory. The majority of these cytokines have been reported to be expressed by the placenta, although expression is highly variable depending on culture conditions, gestational age and disease status [23]–[26]. In addition, we measured levels of tissue inhibitor of metalloproteinases (TIMP)-2, TIMP-4, insulin-like growth factor binding protein (IGFBP)-5, matrix metalloproteinase (MMP)-9, tenascin C, syndecan 1, Fas ligand, osteopontin, TIMP-1, connective tissue growth factor (CTGF), macrophage inflammatory protein (MIP)-1α, MMP-1, IGF-1, MMP-8, monocyte chemotactic protein (MCP)-1, and TIMP-3. These analytes were developed into multiplexed assays due to their importance in schistosomiasis-associated and idiopathic pulmonary fibrosis [27]–[29]. However, they are also widely implicated in invasion and remodeling at the maternal-fetal interface, underscoring the similarities between these two processes.

### Co-culture of pregnant human plasma with HTR8/SVneo cells

For the human plasma assays, we utilized plasma collected from pregnant women at 32 weeks gestation residing in Leyte, the Philippines, an area endemic for *S. japonicum*. The study population and sample collection has been described elsewhere [8], with socioeconomic status (SES), gravida, parity, body mass index (BMI), smoking status and age determined via questionnaire [30]. Schistosomiasis and co-infections (*Ascaris lumbricoides, Trichuris trichuria*, and hookworm) were determined from stool samples using the Kato Katz method. Infection intensities for each were determined using the WHO guidelines [31]. From this larger cohort of 150 women, we selected 29 women infected with schistosomiasis (20 lightly infected [1–99 eggs per gram], 9 moderate infection [100–399 epg]), and 29 uninfected women matched to the infected women for SES, co-infections, gravida, parity, gestational age, BMI, smoking status and maternal age (Table 1). Schistosomiasis was evaluated as a nominal (yes/no) variable due to the low numbers of women with moderate-high infection intensities. Serum was collected at the only pre-natal study visit (32 wks gestation).

**Table 1:** Characteristics of patients from whom plasma was selected for co-culture with HTR8 cells *in vitro*.

	Sj infected (n = 29)	Sj uninfected (n = 29)
*As infections (n)*	21	21
*Tr infections (n)*	23	24
*Hk infections (n)*	10	9
*SES, mean (CI)*	15.2 (13.4, 17.1)	14.9 (13.5, 16.3)
*Smoking status (n)*		
*Yes*	0	0
*No*	29	29
*Gestational age, median (IQR)*	39.3 (38.0, 40.1)	39.0 (38.0, 40.0)
*BMI, median (IQR)*	21.3 (19.9, 24.6)	20.8 (19.4, 23.5)
*Maternal age, mean (CI)*	30.2 (28.0, 32.3)	32.4 (30.2, 34.6)
*Parity, median (IQR)*	2.0 (1.0, 4.0)	3.0 (1.0, 5.0)
*Gravida, median (IQR)*	3.0 (2.0, 5.0)	4.0 (2.5, 6.0)

Sj: *Schistosoma japonicum*, As: *Ascaris lumbrocoides*, Tr: *Trichuris trichuria*, Hk: hookworm, SES: socioeconomic status, BMI: body mass index. Mean (±95% confidence interval) for normally distributed variates; Median (interquartile range) for non-normally distributed variates.

HTR8/SVneo cells were cultured to 80% confluency in complete media before being cultured for 48 hours in serum free media with the addition of 10% plasma from the aforementioned pregnancies. Following 48 h incubation, trophoblast culture supernatants were collected and analyzed for cytokine production as described above.

### Migration assay

HTR8/SVneo cells were cultured to 100% confluence in complete media. Once completely confluent, a scratch was made across the well using a sterile pipette tip. The underside of each of the wells was cross-hatched for reference. Cells were briefly washed with PBS in order to remove all detached cells after scratching, and cultured in serum-free media with the addition of SEA (25 µg/ml) for 48 h. Phase contrast images of the denuded region were taken at 0 h, 24 h and 48 h after scratch formation using an Olympus IX70 inverted tissue culture microscope (Olympus Corp., Tokyo, Japan). The same region of each well was imaged at each time point, using the cross-hatching as reference. The area free of cells was quantified using ImageJ software (NIH, Betheseda, MD). The denuded area at 24 h and 48 h for a specific well was expressed as a percentage of the denuded area that had been present in that well at 0 h, thus controlling for well-to-well variation in original scratch sizes.

### MTT proliferation assay

MTT assays were done on HTR8/SVneo cells in parallel to the migration assays. MTT (Sigma Aldrich, St. Louis, MO) was added to each well and the cells were incubated for 4 h at 37°C in a humidified environment. Media and MTT were aspirated from each well, MTT solvent (4 mM HCl, 0.1% Nonidet P-40, in isopropanol) added, and the plate incubated at 25°C in the dark with rotation for 15 minutes. Absorbance for each well was read at 560 nm and 630 nm.

### Matrigel invasion assay

HTR8/SVneo cells at 80% confluency were treated with SEA (25 µg/ml) for 24 h in complete media before being gently trypsinized, washed with complete media and resuspended in serum-free media. 25,000 cells/well were plated on matrigel-coated transwell inserts with an 8 µm pore size (Corning, Tewksbury, MA). The bottom chamber contained complete HTR8 media. Following 48 h incubation, cells and matrigel were gently removed from the top of the transwell, and those cells that had invaded through the matrigel, traversed the pores, and reached the bottom of the transwell were stained with hematoxylin (Sigma Chemical). Stained cells were visualized and counted using an Olympus BH-2 microscope (Olympus Corp.).

### Statistical analysis

**Figure 1:**
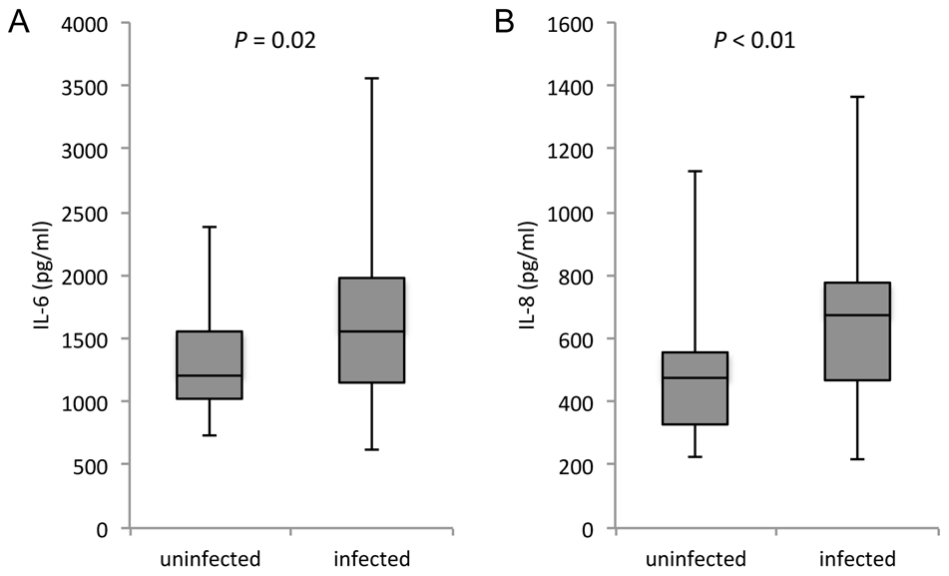
HTR8 cells increase pro-inflammatory cytokine production in response to plasma from schistosome-infected pregnant women. The first-trimester cell line, HTR8/SVneo, was cultured in serum-free media with the addition of 10% plasma from women with schistosomiasis or uninfected women at 32 weeks of gestation. Culture supernatants from cells with cultured with plasma from infected women had higher levels of A) IL-6, *P*<0.02 and B) IL-8. *P*<0.01. n = 58 (29/group). Median and IQR presented.

**Figure 2:**
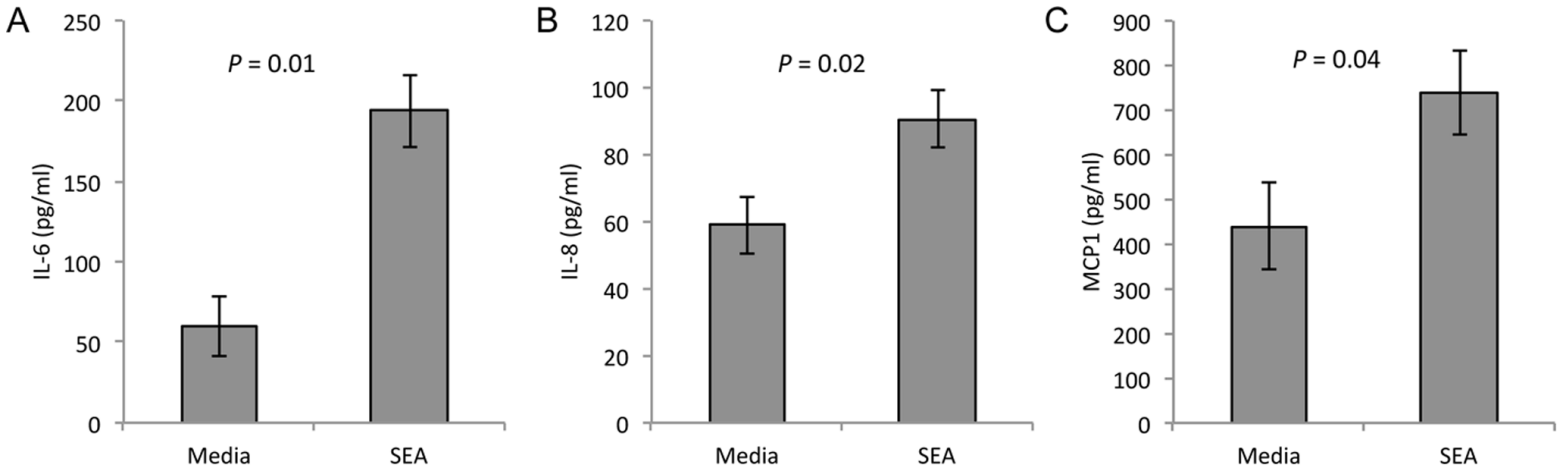
Production of pro-inflammatory cytokines/chemokines are upregulated by SEA in HTR8 cells. The first-trimester cell line model, HTR8/SVneo, was treated with SEA (25 ug/ml) for 24 h. Media from all treatment conditions was collected and measured for cytokine expression. **A and B**) IL-6 & IL-8 production are both increased after SEA exposure. *P* = 0.01 and 0.02, respectively, n = 9 **C**) MCP1 production is increased after SEA treatment. *P*<0.04, n = 13 pairs of HTR8 cells cultured +/− SEA.

**Figure 3:**
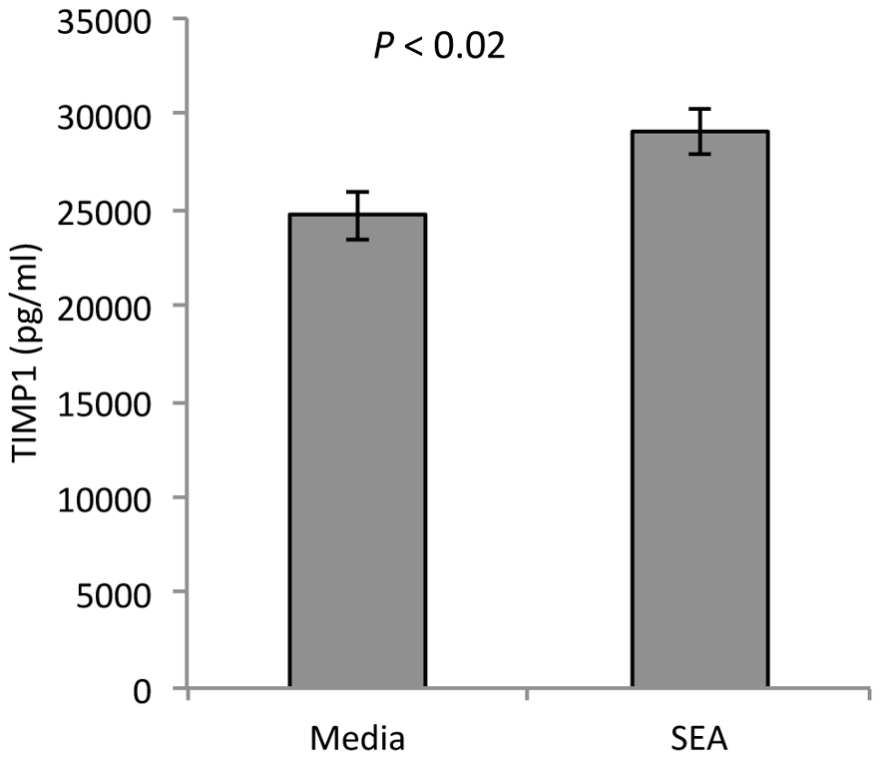
SEA alters the TIMP production in trophoblast cells. The first-trimester cell line model, HTR8/SVneo, was treated with SEA (25 ug/ml) for 24 h. TIMP1 production is increased after SEA treatment. *P*<0.02, n = 13 pairs of HTR8 cultured +/− SEA.

**Figure 4:**
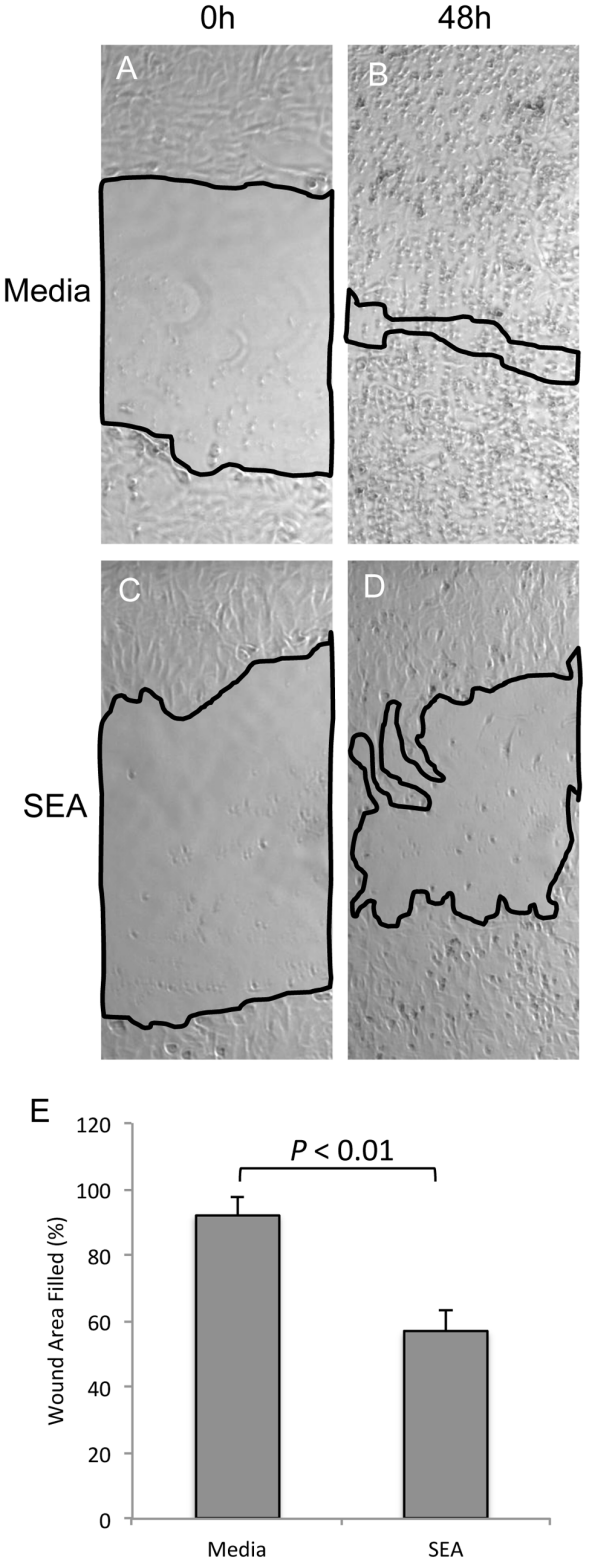
SEA inhibits trophoblast migration. HTR8/SVneo cells were grown to confluence before a linear scratch was made through the center of the growth area. Media was then replaced with either serum-free media alone, or serum-free media with SEA (25 µg/ml) for 48 h. Data for each well was analyzed as the percentage of the original denuded area remaining open at each time point. **A and B**) A well with media alone, at 0 h and 48 h. **C and D**) An area scratched and treated with SEA has little cell migration into the scratched region 48 h later. **E**) The percentage of area filled after 48 h in culture is significantly less in those wells exposed to SEA, *P*<0.01, n = 5 pairs of HTR8 cells cultured +/− SEA.

**Figure 5:**
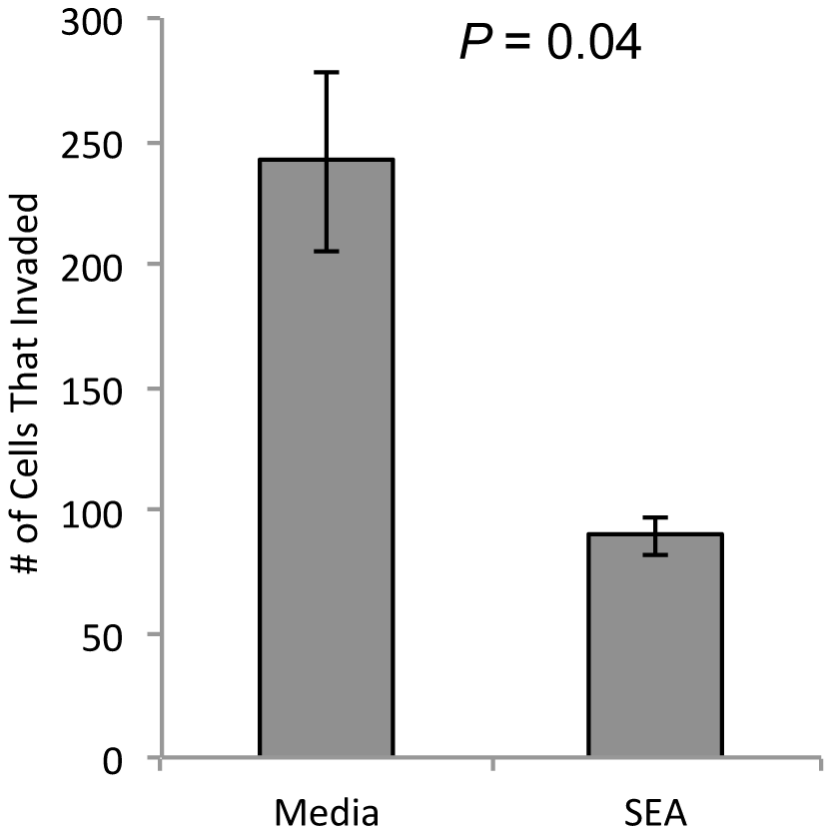
SEA inhibits invasion of HTR8 cells. The first-trimester cell line, HTR8/SVneo, was treated with SEA (25 ug/ml) for 24 h, after which they were plated in matrigel coated transwell inserts. Fewer cells reached the bottom of the transwell when previously exposed to SEA. *P* = 0.04, n = 5 pairs of HTR8 cultures +/− SEA.

Data analysis was performed using JMP v.10 (SAS Institute, Cary, NC). All data were evaluated for normality using the Shapiro-Wilk test. Those experiments for which all data were normally distributed were further evaluated with ANOVA and t-tests, with means ± SEM reported. For data that was not normally distributed, Wilcoxon Signed Rank analyses were performed, with data reported as median ± IQR. Specifically, cytokine production by HTR8 cells was compared between cells exposed to uninfected plasma and those exposed to infected plasma (Figure 1) as well as cells cultured with media alone and media with SEA (Figures 2 and 3). Similarly, HTR8 migration and invasion were compared between cells cultured with media alone and those with SEA addition to the media (Figures 4 and 5). Statistical significance was considered as *P*<0.05.

## Results

### Plasma from pregnant women infected with schistosomiasis causes increased pro-inflammatory cytokine production in first trimester trophoblast cells

For these experiments, we selected a sub-set of plasma samples collected from women at 32 weeks gestation as part of a previous study [8], matching samples from 29 infected women with 29 samples from uninfected women on key potential confounding covariates (Table 1). HTR8/SVneo cells were cultured in serum-free media supplemented with 10% plasma collected at 32 weeks gestation, and allowed to remain in culture for 48 h. Media from HTR8/SVneo cells cultured with plasma from schistosome-infected women had significantly higher levels of the pro-inflammatory cytokines IL-6 and IL-8 (29%, *P*<0.02 and 42%, *P*<0.01, respectively), compared with cells cultured in the presence of plasma from uninfected pregnant women (Fig. 1). Levels of both IL-6 and IL-8 in the plasma alone were in most cases undetectable, with the highest level of either cytokine across all plasma samples being 32 pg/ml (data not shown). These data indicate that schistosomiasis results in the production of some factor(s), present in maternal circulation, that stimulate a pro-inflammatory cytokine response by first trimester trophoblasts.

### Schistosome soluble egg antigens generate a pro-inflammatory response in HTR8 cells

Given that SEA are found in the circulation of infected individuals and are known to cross the placental barrier, we treated HTR8/SVneo cells with purified SEA (25 µg/ml) in culture. Within 24 h of culture, HTR8 cells treated with SEA secreted higher levels of IL-6 (3.2-fold, *P* = 0.01), IL-8 (1.5-fold, *P* = 0.02) and the pro-inflammatory chemokine, MCP-1 (also known as CCL2; 1.7-fold, *P* 0.04) into the culture media, as compared to HTR8/SVneo treated with media alone (Fig. 2). Not only do first trimester cell models respond to SEA with a pro-inflammatory signature, they also secrete higher levels of a chemo-attractant protein that may help recruit specific immune cells to the placenta, exacerbating the inflammatory reaction initiated by SEA at the maternal-fetal interface.

### Extracellular matrix remodeling proteins are altered in HTR8 cells after treatment with SEA

In addition to cytokine analysis, media from HTR8/SVneo cells treated with SEA for 24 h in culture were assessed for altered levels of a number of fibrotic markers. Of these, TIMP-1 production was increased in media from HTR8 treated with SEA, compared to media from cells with no SEA exposure (Fig. 3). In contrast, production of CTGF, CCL18, TIMP-3 and fibronectin all showed no difference in levels secreted from HTR8/SVneo cells exposed to SEA compared to those with media alone (data not shown). Given the important role of TIMP-1 in the inhibition of MMPs, SEA may influence the ability of these first trimester cells to remodel and migrate into the maternal uterine wall.

### HTR8 cells have decreased migration after exposure to SEA in culture

We performed *in vitro* wound assays to assess migration of the first trimester cell line, HTR8/SVneo, following exposure to SEA. There was little difference in cell migration at 24 h (Fig. 4a). By 48 h however, HTR8 cells in culture with SEA had filled only 57±8% of the denuded area compared to 92±6% for untreated cells (*P*<0.01, Fig. 4). Cell proliferation was measured using MTT assay in all wells, however no differences were observed between those wells with SEA added compared to those with media alone indicating the wound closure was not due to a proliferative effect of SEA treatment (data not shown). Together, these data suggest that migration of first trimester trophoblast cells is decreased in the presence of SEA.

### Invasion of HTR8 cells through matrigel is inhibited by SEA

We next assessed the ability of the HTR8/SVneo cell line to invade through matrigel, a model of extracellular matrix, and a transwell insert after being treated with SEA, using a standard invasion assay [32]. Cells were pretreated with SEA for 24 h to minimize any direct effect of SEA on the matrigel. Enumeration of the cells that had traversed the matrigel and the pores of the transwell after an additional 48 h incubation showed 2.7-fold lower absolute cell numbers in those wells that had been pre-treated with SEA compared to the HTR8 cells that received media alone for the initial 24 h (*P* = 0.04, Fig. 5). These data indicate that SEA inhibits the migratory and invasive properties of the first trimester cell line, HTR8/SVneo.

## Discussion

Despite a 2002 WHO recommendation that pregnant and lactating women be considered for inclusion in treatment programs [33], pregnant women with schistosomiasis are still excluded in many regions pending further evaluation of praziquantel's safety during pregnancy. Data regarding the impact of schistosome infection on human pregnancy is rather scant, although we have reported an increase in pro-inflammatory markers in maternal, placental, and newborn compartments of pregnancies complicated by schistosomiasis, as well as increased rates of acute subchorionitis in these women [8]. Several human studies have evaluated the role of schistosomiasis during pregnancy [11], [34], [35]. Two observational studies reported lower birth weights among infants from infected mothers. However, methodological issues, including lack of control for important potential confounders such as socioeconomic status and maternal nutritional status [35], and potential selection bias [11] make interpretation difficult. A recently completed RCT evaluating treatment of schistosomiasis during pregnancy in Uganda reported no change in birth weight among women treated for schistosomiasis during the second trimester and untreated controls [34]. Any effect(s) of schistosomiasis on early placentation however would not be detected because treatment occurred late in gestation. Thus, questions regarding the influence of schistosome infection during the first trimester of pregnancy in humans remain largely unanswered.

Although direct trafficking of the adult worm or schistosome eggs to the maternal-fetal interface is thought to be a rare event [9], schistosomiasis is known to produce a distinct antigenic signature in the circulation of infected individuals, including the presence of high levels of soluble egg antigens (SEA) which can cross the placental barrier [12]. Previously, we demonstrated that SEA can cause pro-inflammatory cytokine production in primary human trophoblast cells taken at term and allowed to syncytialize *in vitro* [19]. However, placentation is a dynamic process requiring trophoblast populations distinct in time and differentiation lineage to behave in specific and unique ways. In this manner, a syncytialized term trophoblast is responsible for very different functions (i.e. nutrient transfer, cytokine and hormone production) than a first trimester invasive trophoblast cell. In this report, we have focused on the effect of SEA on first trimester trophoblast cells, using the cell line HTR8/SVneo, as these cells represent one of the best model systems available for studying the behavior and characteristics of invasive, extravillous trophoblast cells [36].

As we have previously reported in term syncytialized trophoblasts, HTR8 cells exposed to SEA for 24 h (25 µg/ml) exhibited a pro-inflammatory cytokine signature. These findings echoed the pro-inflammatory signature we observed in HTR8 cells exposed to plasma from pregnant women infected with schistosomiasis, compared to plasma from pregnant, uninfected controls. A potential limitation of these experiments is that HTR8 cells were cultured with maternal plasma collected during the third trimester because the original study from which these samples originated did not enroll pregnant women until 32 weeks gestation [8]. We do not expect this to influence either the validity or generalizability of our results as schistosomiasis infection status and its consequent host response should not be altered during gestation.

Although little is known regarding the impact of localized pro-inflammatory cytokines during the first trimester, they have been suggested to contribute to reduced migration and invasion of trophoblast cells, increased migration of innate immune cells to the maternal fetal interface, and, at very high levels, are postulated to play a role in preterm delivery and/or miscarriage [21], [37], [38].

Another major role of extravillous trophoblast cells, particularly in the first trimester, is to remodel and invade deep into the maternal endometrium. This process is tightly regulated, and failure to invade to the appropriate degree has been associated with the development of a number of gestational diseases, most importantly preeclampsia, preterm birth and low birth weight [39], [40]. Failure to identify any difference in the cellular metabolic activity (MTT assay as a surrogate for cell proliferation) between the untreated and SEA-exposed cells supports the idea that SEA is not simply cytotoxic to HTR8 cells. Rather, HTR8 cells display increased production of TIMP-1, inhibition of cellular migration and decreased levels of invasion through matrigel when exposed to SEA. Of the fibrosis-associated molecules measured, TIMP-1 is arguably the most relevant to trophoblast migration/invasion [41], [42]. These data suggest that the process of placentation could be compromised in pregnancies complicated by schistosomiasis during the first trimester. To our knowledge, there have been no studies examining the effect, if any, of schistosomiasis on gestational diseases such as preeclampsia.

Our data are consistent with previous work from our laboratory regarding schistosome induced pro-inflammatory cytokine production across different models of trophoblast cells [19]. Outside of pregnancy, we and others have related these responses to nutritional, hepatic and hematologic morbidities in infected individuals [43]–[46]. Surprisingly, maternal schistosomiasis during pregnancy elicits a pro-inflammatory response detectable in the neonate [8], and neonates exposed to maternal schistosomiasis during pregnancy display a more robust response to antigenic challenge and have elevated levels of antibodies against schistosome antigens at birth than their unexposed counterparts [13], [47]. Together, these results suggest that maternal schistosomiasis may influence the outcome of initial pediatric schistosome infections acquired during early childhood.

The finding that SEA may modify invasion of extravillous trophoblasts and alter the cytokine milieu at the maternal fetal interface lends support to an aggressive treatment approach for women of reproductive age, such that they enter pregnancies infection free. It should also be noted that studies which have, and will, evaluate the efficacy of praziquantel given after the first trimester (ClinicalTrials.gov, registered study number NCT00486863), may not capture the full benefit of treatment as it relates to early placentation processes. Studies regarding the incidence of gestational diseases such as preeclampsia in the context of high schistosome prevalence are warranted and may shed additional light on the impact of schistosomiasis on the early development of the human placenta.
